# Thermally Activated Stress Relaxation and Creep in Ideal Hydrogel Elastomers: Rupture of Tensile Strands

**DOI:** 10.1021/acspolymersau.6c00040

**Published:** 2026-04-20

**Authors:** Chih-Jung Lin, Yi-Cheng Kao, Heng-Kwong Tsao, Yu-Jane Sheng

**Affiliations:** † Department of Chemical Engineering, National Taiwan University, Taipei 106, Taiwan; ‡ Department of Chemical and Materials Engineering, 34911 National Central University, Chung-li, Taoyuan 320, Taiwan

**Keywords:** microscopic mechanism, hydrogel, stress relaxation, creep, bond rupture, dissipative particle dynamics

## Abstract

The microscopic origins of time-dependent mechanical responses, including stress relaxation and creep, in a hydrogel film composed of a chemically cross-linked network are investigated using dissipative particle dynamics. After free relaxation following uniaxial stretching, the model hydrogel exhibits full recovery at moderate strains but develops a small residual strain at larger deformations, indicating an elastomeric behavior accompanied by irreversible plasticity. Microscopic analyses reveal the evolution of the bond length, strand length, internal energy, strand stretch ratio, and bond rupture during deformation and relaxation. Stress relaxation and creep are essentially absent at moderate strains, consistent with negligible frictional dissipation, but become pronounced at larger strains due to the occurrence of rare bond-rupture events. During stress relaxation, the decay of stress closely correlates with reductions in the bond length and internal energy with the response dominated by bond-length relaxation associated with network reorganization. During creep, the mean bond length remains nearly constant, whereas the mean strand length evolves through primary/secondary regimes and accelerates into tertiary creep-to-failure. Both time-dependent behaviors arise from structural reorganization induced by rare thermally activated bond-rupture events localized within highly stretched tensile strands.

## Graphical abstract

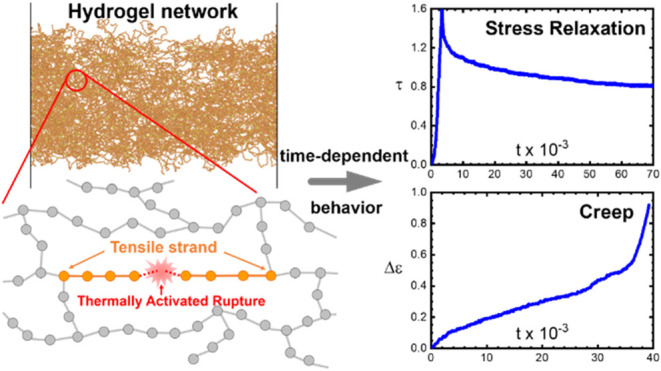

## 1 Introduction

Hydrogels are three-dimensional (3D) networks of hydrophilic polymers swollen with water, with their softness and deformability arising from the high solvent content.^1–3^ Despite their intrinsic softness, some hydrogels can exhibit elastomer-like elasticity. A representative example is elastin-like polypeptide hydrogels,^4,5^ which are widely used in applications ranging from tissue engineering to soft robotics.^6,7^ When elastomers are subjected to a deformation held at a fixed strain, the stress gradually decreases with time and eventually approaches a plateau, a behavior known as stress relaxation.^8,9^ When subjected to a constant applied stress, elastomers may undergo progressive deformation over time, referred to as creep. These time-dependent responses are generally attributed to the viscoelastic nature of the elastomers. By contrast, elastomers such as natural rubber and silicone can undergo large, reversible deformations with minimal residual strain,^10,11^ and their time-dependent responses, including stress relaxation and creep, are negligible. Therefore, they can be regarded as nearly ideal elastomers under moderate deformation.

In an ideal elastic solid held at a fixed strain, the stress would remain constant over time, implying that structural evolution follows the equilibrium path and that deformation upon strain change is essentially reversible. In such a system, friction-induced irreversibility, commonly observed between polymer chains, is negligible, and no further re-equilibration is required after the target strain is reached. By contrast, time-dependent stress decay signifies a deviation from ideal elasticity and is generally attributed to frictional dissipation and structural reorganization in polymer systems.^12–18^ During elongation, polymer chains at entanglements are forced to stretch and slide past one another, generating viscoelastic dissipation that requires additional work to overcome. As a result, a stress higher than that along the equilibrium path is required to sustain a given strain. When a stretched, nonequilibrium polymer system is held at a fixed strain, the polymer chains relax through conformational rearrangements and structural reorganization, evolving toward their equilibrium state with a lower internal stress.^19,20^ The relaxation amplitude and time scale provide sensitive measures of how far the polymer system is from its equilibrium state, which is directly relevant to the long-term mechanical stability of devices.^8,21^

In addition to stress relaxation, creep tests also provide an assessment of the long-term durability of polymer materials under sustained, constant loads. The creep response of polymer systems typically proceeds through three distinct regimes.^22^ During the primary creep stage, the strain increases, while the strain rate gradually decreases. This is followed by secondary creep, a quasi-steady-state regime in which the strain rate remains approximately constant. When sufficiently high stresses are applied, the material enters tertiary creep, characterized by a rapid acceleration of the strain rate that ultimately leads to macroscopic failure.^8,23–25^ At a fixed applied stress, the system at the initially reached strain is in a nonequilibrium state and tends to evolve toward equilibrium through structural rearrangement.^19,26–28^ As a result, similar to stress relaxation, the internal stress decreases as the frictional contribution generated during stretching vanishes and can no longer balance the external load, leading to further elongation of the polymer system. To sustain the constant external load, the strain continues to increase in order to reduce the mismatch between the internal stress and applied load.

In highly cross-linked hydrogels, chain mobility through slippage and disentanglement is strongly suppressed, such that viscoelastic effects are expected to be limited; nevertheless, pronounced stress relaxation and creep behavior can still be observed.^8,23^ Bond rupture is proposed as a key contributor to stress relaxation, yet the microscopic-to-macroscopic pathway by which such events produce pronounced mechanical changes remains unclear. Deterministic bond rupture is generally assumed to occur only during stretching and is not expected to occur during stress relaxation, during which the deformation (strain) remains unchanged. If rupture were purely mechanical, stress relaxation under a fixed strain would be minimal because intact bonds would be unlikely to exceed the critical bond length spontaneously. To overcome the experimental challenge of resolving microscopic dynamics, we employ coarse-grained simulations of a hydrogel elastomer in which bonds are permitted to break once sufficiently stretched. Our recent simulations indicate that force-assisted, rare, thermally activated bond rupture can drive stress relaxation.^29^ However, the spatiotemporal features of rupture, specifically, when it occurs, where it localizes under load, and how such rare events give rise to macroscopic time-dependent mechanical responses, remain poorly understood. The present work aims to elucidate a unifying microscopic mechanism in which thermally activated bond rupture within highly stretched tensile strands governs both stress relaxation and creep via the reconfiguration of the load-bearing network.

## 2 Methods

### 2.1 Interaction Forces and Polymer Networks

Dissipative particle dynamics (DPD) is a mesoscale simulation technique widely used to model polymer networks and soft-matter systems.^30–32^ In DPD, the system is represented by interacting beads, each corresponding to a coarse-grained group of atoms or molecules, whose dynamics are governed by Newton’s equations of motion. Compared with conventional molecular dynamics, DPD enables access to larger time and length scales through its coarse-grained description and soft interaction potentials. The interaction between a pair of beads comprises three short-ranged, pairwise additive contributions: a conservative force (*f*_*ij*_^*C*^), a dissipative force (*f*_*ij*_^*D*^), and a random force (*f*_*ij*_^*R*^).^30,33^ Accordingly, the total force acting on bead *i* is obtained by summing these contributions over all neighboring beads *j* within the cutoff radius *r*_c_, beyond which all DPD interactions vanish (*r*_ij_ > *r*_c_). The conservative force captures bead–bead repulsion and is given by *f*_*ij*_^*C*^ = *a*_*ij*_(1 – *r*_*ij*_/*r*_c_)*r̂*_*ij*_, *r*_*ij*_ < *r*_c_, with *a*_*ij*_ being the repulsion parameter, *r*_*ij*_ being the particle distance, and *r̂*_*ij*_ being the unit vector pointing from bead *j* to bead *i*. The dissipative force acts as a velocity-dependent friction that dampens relative motion, whereas the random force represents thermal fluctuations. Note that the degree of coarse-graining does not affect the thermal fluctuations (*k*_B_ *T*) inherent in DPD. Together, *f*_*ij*_^*D*^ and *f*_*ij*_^*R*^ are constructed to satisfy the fluctuation–dissipation theorem and maintain an isothermal condition,^34^ while momentum conservation is ensured by Newton’s third law.

In DPD simulations, all physical quantities are nondimensionalized using bead mass *m*, cutoff radius *r*_c_, and thermal energy *k*_B_ *T*. For example, the simulation time can be scaled by $\sqrt{mr_{c}^{2}/k_{B}T}$ , while stress is scaled by *k*_B_ *T*/*r*_c_^3^. A bead number density of ρ = 3 per unit volume is adopted, which is commonly used in DPD simulations and yields a reasonable effective compressibility for water-like solvents.^30,35^ The mapping between a DPD bead and the underlying chemical structure is system-dependent, allowing each bead to represent multiple atoms or molecules as needed.^32,36^ For instance, in DPD studies of polyacrylamide (PAAm) hydrogels, a common mapping assigns one bead to two AAm repeat units, whereas one solvent bead corresponds to eight water molecules.^37,38^ All DPD simulations were performed using the open-source package LAMMPS.^39^ The integration time step was set to Δ*t* = 0.01, and the temperature was maintained at *T* = 1 throughout.

In this work, we employ a model of a chemically cross-linked hydrogel film to investigate stress relaxation and creep tests. The network is generated by cross-linking polymer chains with cross-linkers and subsequently swelling the structure in a good solvent. Following our previous study,^29,32^ each polymer is modeled as a linear chain of 120 DPD beads. Three bead types are considered: polymer, cross-linker (dimer), and solvent. Beads along each polymer chain or cross-linker are connected by harmonic springs, described by the force *f*_*ij*_^*S*^ = *k*_s_(*r*_*ij*_ – *r*_0_)*r̂*_*ij*_, where *k*_s_ = 300 and *r*_0_ = 0.4. The equilibrium bond length is chosen to be much smaller than the bead size to suppress chain crossing, thereby maintaining polymer topological constraints and more accurately representing the real polymer system.^40^ Chain stiffness is introduced through an angular potential applied to successive beads along each polymer chain, *U* ^θ^ = *k*_θ_(θ – θ_0_)^2^, where *k*_θ_ = 0.5 for intrapolymer angular interactions and θ_0_ = π, corresponding to semiflexible chains. In contrast, angular potentials involving cross-linkers are assigned *k*_θ_ = 0, allowing free bending. Nonbonded interactions between directly bonded beads are excluded to minimize the mean bond length. Bond rupture is permitted when *r*_ij_ > *r*_b_ = 0.8, which corresponds to a bond energy of 24 *k*_B_ *T*.

This bond dissociation energy is lower than the typical energy barrier of a covalent bond (50–100 *k*_B_ *T*) but is comparable to that of dynamic covalent bonds (15–60 *k*_B_ *T*). The relatively low energy barrier was chosen to enable bond rupture within the simulation time scale rather than the much longer time scale of real experiments. Moreover, the bond in the present DPD model is defined in an effective coarse-grained sense rather than as a direct representation of a real bond between two individual atoms. Since each DPD bead represents a group of atoms or molecules, the bond corresponds to an effective load-bearing connection between coarse-grained segments in the network. The rupture events are intended to represent scission processes analogous to chemical bond breakage in real networks. Therefore, this rupture criterion should be interpreted not as the scission energy of a specific covalent bond but as an effective activation barrier for the loss of load-bearing connectivity in the network, which is lower than the typical bond dissociation energy.

To provide physical context for the reduced DPD units, an illustrative mapping may be made to representative elastin-like pentapeptide (ELP)-based hydrogels. Reported ELP chains typically contain tens to hundreds of pentapeptide repeats. If one DPD bead is taken to represent approximately five ELP repeats, then the average strand length in the present network, ∼10 beads, corresponds to a cross-link-to-cross-link strand length of ∼50 pentapeptide repeats. Using a pentapeptide molecular mass of approximately 420 g/mol and a contour length of approximately 1.8 nm gives *m* ≈ 3.49 × 10^–24^ kg and *r*_c_ ≈ 9.0 nm in our simulation. The corresponding stress scale is *k*_B_ *T*/*r*_c_^3^ ≈ 5.6 kPa at room temperature. This illustrative mapping is used to place the nondimensional DPD results in the context of representative ELP-based hydrogels and to provide a comparison with available experimental data and the expected material range.^40–43^

### 2.2 Simulation System and Equilibration

The simulation system follows our previous setup,^29,32^ in which a hydrogel film is placed between two solvent baths. Accordingly, the hydrogel remains fully immersed in the solvent throughout the simulation, as schematically illustrated in Figure S1. The hydrogel film contains 22.5 wt % polymer, 2.5 wt % cross-linker, and 75 wt % solvent, corresponding to a total of 332,640 beads at a number density of ρ = 3. Identical repulsive parameters *a*_*ij*_ = 25 are applied to all bead pairs, indicating good mutual compatibility.^30^ Periodic boundary conditions are imposed in all three directions. To generate a well-relaxed initial state, the system is equilibrated by using a two-step procedure. In the first step, equilibration is performed under isothermal and isochoric conditions (NVT ensemble) with the simulation box fixed at 33.3 × 33.3 × 99.9. Note that because the network is formed under the NVT ensemble, residual stresses remain in the initial configuration. The second step is carried out under isothermal and isobaric conditions (NPT ensemble). Allowing the box dimensions to fluctuate effectively relieves residual stresses imposed by the previous fixed-volume constraint. After NPT relaxation, the box slightly contracts in the *x* and *y* directions and elongates along the *z* direction. Despite these shape changes, the total volume and number density remain nearly unchanged. The resulting configuration is used as the initial state for all subsequent deformation simulations. Because of the thin-film geometry, anisotropy arises naturally and the gyration tensor of the polymer chains exhibits comparable *xx* and *yy* components, with a smaller *zz* component.

### 2.3 Relaxation Protocols and Time-Dependent Responses

The mechanical response of the hydrogel is examined using the ‘fix deform’ command in LAMMPS under the NVT ensemble. The simulation box is continuously stretched along the loading direction until a prescribed strain is reached, thereby imposing uniaxial deformation. Under periodic boundary conditions, the loading is imposed by deforming the simulation box along the *x* direction. The box length along the loading direction evolves as *L*_*x*_(*t*) = *L*_*x*0_(1 + ε̇*t*), where *L*_*x*0_ is the initial box length and ε̇ is the strain rate. The engineering strain is defined as ε = (*L*_*x*_ – *L*_*x*0_)/*L*_*x*0_, and the corresponding stretch ratio is given by λ = *L*_*x*_/*L*_*x*0_ = 1 + ε. The instantaneous stress is calculated as τ = τ_*xx*_ – (τ_*yy*_ + τ_*zz*_)/2,^44,45^ where τ_kk_ represents the average virial stress component in direction k, defined as negative of the corresponding diagonal element of the pressure tensor *P*_*kk*_. Instead of the loading-direction normal stress itself, τ_*xx*_, τ represents the elongational stress and corresponds to the axial deviatoric stress, i.e., the tensile component of the stress with the hydrostatic contribution removed. The pressure tensor contains both kinetic and virial contributions and is given by $P_{\mathrm{kk}}=\sum_{i}m^{i}v_{k}^{i}v_{k}^{i}/V+\sum_{\left(j\neq i\right)}f_{k}^{\mathrm{ij}}r_{k}^{\mathrm{ij}}/2V$ , where *V* denotes the volume of the simulation box, *m* ^*i*^ and *v*_*k*_^*i*^ are the mass and velocity components of particle *i*, and *f*_*k*_^*ij*^ and *r*_*k*_^*ij*^ are the force and distance components between particles *i* and *j*, respectively.^46,47^ Note that a pure solvent system exhibits zero tensile stress upon deformation.

Free-relaxation simulations are carried out in the NPT ensemble to examine the spontaneous recovery of the hydrogel from a uniaxially stretched state. To accurately capture the relaxation dynamics, the pressure damping constant is set to 50 to suppress rapid pressure fluctuations. During free relaxation, the target pressure is set to the equilibrium value of the unstretched system. Upon releasing the applied load from a specified strain, the system relaxes and tends to restore its original shape and volume. Such coupled stress–strain evolution, occurring without any external constraint during free relaxation, is difficult to measure experimentally. Unlike free relaxation, stress relaxation is characterized by the gradual decay of internal stress under a fixed strain. In practice, once the target strain is reached during uniaxial deformation, the simulation box dimensions are held fixed in the NVT ensemble to maintain a constant strain condition, and the time-dependent stress response is monitored to quantify the relaxation amplitude.

In contrast to stress relaxation, a creep test probes the strain increase under constant applied stress. In simulations, the constant-stress condition can be maintained by controlling the simulation box dimensions through a barostat in the NPT ensemble. Specifically, the target stress component is imposed via the pressure tensor, allowing the simulation cell to deform in response to the applied load. In LAMMPS, this is implemented using the “fix npt” command with anisotropic pressure control, where the target stresses (e.g., *P*_*xx*_, *P*_*yy*_, and *P*_*zz*_) are set to desired constant values. The barostat then continuously adjusts the box dimensions to maintain the imposed stress, providing a constant-stress boundary condition analogous to experimental creep measurements and enabling direct monitoring of the strain evolution over time. To ensure statistical reliability, all reported mechanical responses are averaged over at least ten independently equilibrated simulations with identical parameters but different initial configurations.

## 3 Results and Discussion

The macroscopic mechanical response and microscopic mechanisms of an ideal hydrogel elastomer are investigated under uniaxial extension. A thin film of a hydrogel elastomer, consisting of 22.5% polymer, 75% solvent, and 2.5% cross-linker, immersed in the same good solvent is considered. The mean film thickness is 7.50 *R*_g_, which is substantially larger than the radius of gyration of a polymer chain freely suspended in the solvent (*R*_g_ = 3.90). In Section 3.1, the stress–strain curve of the hydrogel elastomer is presented, showing the occurrence of necking and progression to the final fracture stage. The elastomer’s ideality is verified through free relaxation, and the contribution of friction is assessed. In Sections 3.2 and 3.3, stress relaxation is examined, where microstructural rearrangements are revealed through changes in the internal energy. The microscopic mechanism underlying stress relaxation is attributed to the infrequent rupture of strands and bonds. In Section 3.4, the creep test, in contrast to stress relaxation, is also explored, and the microscopic mechanisms associated with its three stages are elucidated.

### 3.1 Stress–Strain Curve and Free Relaxation

The mechanical behavior of the hydrogel film can be characterized by its stress–strain curve under uniaxial stretching at a constant strain rate of ε̇ = 5 × 10^–4^. Figure 1 illustrates the variation in the average stress (τ) with engineering strain (ε) up to the fracture point, where the maximum stress corresponds to the tensile strength. A supplementary comparison of τ as a function of the stretch ratio (λ) is provided in Figure S2. The point at which the stress drops to zero represents the elongation at break. The insets show the morphological changes of the hydrogel during the entire stretching process. With increasing strain, the film thickness decreases uniformly until necking develops after the tensile strength is surpassed. Subsequently, the film bulges outside the necking region, where thin filaments bridge the two bulging domains, ultimately leading to rupture. This mechanical response is characteristic of typical hydrogels. From the stress–strain curve, the mechanical properties of the film are determined, yielding a Young’s modulus of 0.212, a tensile strength of 1.67, an elongation at break of 3.72, and a toughness of 2.71. Under the illustrative ELP-based mapping introduced in Section 2.1, these values correspond to a Young’s modulus of ∼1.19 kPa, a tensile strength of ∼9.35 kPa, and an elongation at break of 372%, which fall within the experimentally reported range for several ELP-based hydrogels.^40–43^

**Figure 1:**
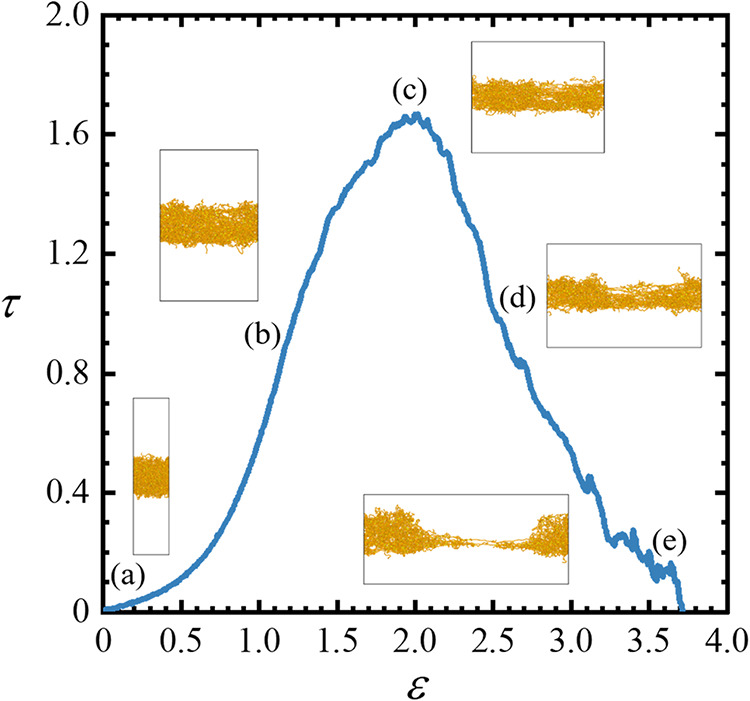
Stress–strain curve of the hydrogel elastomer, with insets illustrating (a) ε = 0 (initial state), (b) ε = 1 (uniform stretching), (c) ε = 2 (tensile strength), (d) ε = 2.5 (necking), and (e) ε = 3.7 (fracture).

To determine whether the hydrogel exhibits elastomeric characteristics, a free-relaxation simulation was performed under isothermal and isobaric conditions (NPT ensemble). Free relaxation refers to spontaneous recovery after unloading, with neither strain nor stress externally controlled. This free-relaxation protocol is distinct from the fixed-strain stress relaxation protocol discussed in Section 3.2. Note that the pressure damping constant in the NPT simulation is set to 50, preventing rapid pressure fluctuations that might interfere with the observations. The free-relaxation behavior after stretching to ε = 0.6 and 1.5 is represented in the corresponding strain–time relationships and stress–strain curves. As shown in Figure 2(a), the sudden release of deformation (ε = 0.6, obtained under the NVT ensemble) leads the strain to relax toward the zero-strain state (ε = 0.0). After a relaxation time of 13,000, only minor thermal fluctuations in strain remain, while the mean value stays effectively zero. The inset of Figure 2(a) shows that as ε decreases, the stress continuously decays and eventually reaches a zero-stress state. Moreover, the loading and free-relaxation (unloading) paths nearly coincide, suggesting that viscous dissipation associated with chain slippage and disentanglement is negligible. These results indicate that a moderate strain (ε = 0.6) within the elastic regime enables reversible structural recovery of the hydrogel, allowing the system to fully return to its initial equilibrium state.

**Figure 2:**
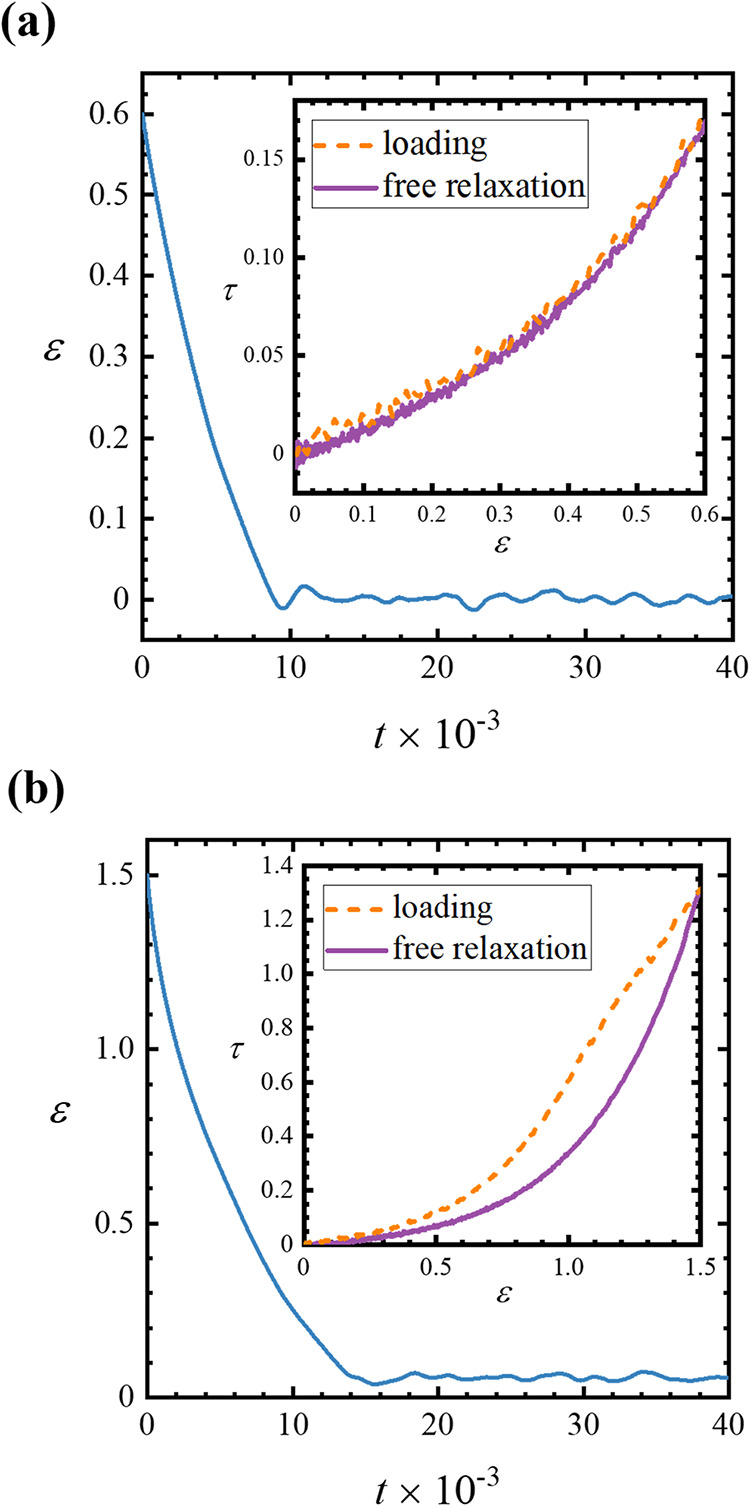
Free relaxation following stretching to (a) ε = 0.6 and (b) ε = 1.5. The strain–time and stress–strain (inset) responses show complete recovery to the zero-strain state at ε = 0.6, whereas relaxation at ε = 1.5 leads to a small residual strain.

For comparison, Figure 2(b) presents the free relaxation following a larger strain (ε = 1.5). Similar to the previous case, the stress decreases with decreasing ε, and it ultimately reaches a zero-stress state. However, in this case, ε decreases exponentially and stabilizes at a small residual strain (∼ε_r_ = 0.08) after a relaxation time of 14,000, corresponding to the zero-stress state but failing to return to the zero-strain state. Unlike the case of ε = 0.6, a hysteresis loop appears between the loading and free-relaxation paths, as shown in the inset of Figure 2(b). The hysteresis loop is generally attributed to mechanical energy dissipation or structural rearrangement during the relaxation process. Evidently, after free relaxation, the hydrogel exhibits elastic behavior, fully recovering to the zero-strain state, at small to moderate strains (e.g., ε = 0.6), but shows plastic behavior, characterized by a residual strain, at large strains (e.g., ε = 1.5). The above analysis demonstrates that the hydrogel can be regarded as an ideal reversible elastomer with insignificant dissipation effects leading to negligible hysteresis behavior at strains below the moderate level. In contrast, within the plastic regime (i.e., at large strains), a residual strain is observed after relaxation, and the typical hysteresis behavior reappears.

In addition to the macroscopic mechanical behavior, the microscopic response of the hydrogel can be elucidated by examining the variation in the mean bond length (Δ*l*_b_) relative to its equilibrium value. Figure 3 shows the evolution of the mean bond-length change, Δ*l*_b_(*t*), during the loading process and subsequent free relaxation. Following deformation to ε = 1.5, Δ*l*_b_ gradually decreases during relaxation and eventually stabilizes near its equilibrium level prior to stretching; the inset shows a relaxation behavior similar to that for ε = 0.6. In both cases (ε = 1.5 and ε = 0.6), Δ*l*_b_ converges to zero after relaxation, accompanied by minor thermal fluctuations around the equilibrium level (Δ*l*_b_ = 0), indicating that the microscopic bond configuration is fully restored to the equilibrium zero-stress state. Notably, the loading and free-relaxation paths of Δ*l*_b_ nearly coincide at ε = 0.6, whereas at ε = 1.5, they enclose a finite area, forming a hysteresis loop. Interestingly, the relaxation behavior of the microscopic property (Δ*l*_b_–ε plot) is analogous to the macroscopic stress response (τ–ε plot): negligible hysteresis at moderate strains but pronounced hysteresis at large strains, indicating that they are closely correlated. Since microscopic hysteresis does not involve energy dissipation, it must arise from structural rearrangements. Consequently, in an ideal elastomer, macroscopic hysteresis observed at large strains is likewise expected to originate from structural rearrangements rather than from energy dissipation.

**Figure 3:**
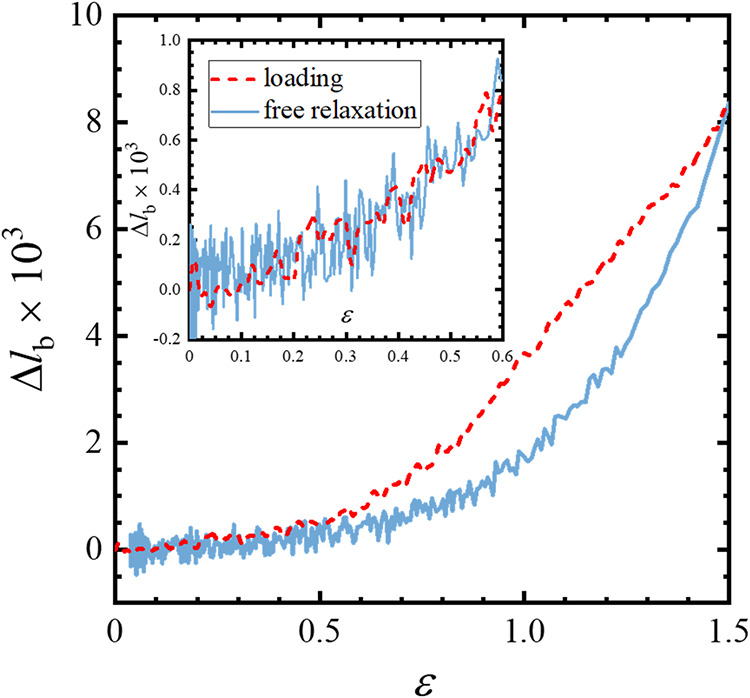
Evolution of the mean bond-length change (Δ*l*_b_) during the loading process and subsequent free relaxation at ε_max_ = 1.5; the corresponding result for ε_max_ = 0.6 is shown in the inset.

### 3.2 Stress Relaxation: Mean Strand and Bond Lengths

In addition to free relaxation, stress relaxation was performed by holding the hydrogel under a constant strain for a prescribed period. Figure 4(a) shows the stress relaxation behavior of the hydrogel elastomer at two strain levels: ε_max_ = 0.7 (moderate strain) and ε_max_ = 1.7 (large strain). Additional stress relaxation curves at multiple imposed strains, with time measured from the onset of stress relaxation, are provided in Figure S3 to help identify the strain threshold above which appreciable relaxation becomes visible. This threshold is found to be around ε ≈ 0.9, corresponding to the onset of bond-rupture-induced plasticity in the present system. When the strain is maintained at ε_max_ = 0.7, the stress remains nearly constant throughout the relaxation period, exhibiting only minor fluctuations around its steady value. This time-independent response indicates that the hydrogel network behaves as an ideal elastomer with negligible viscous dissipation and minimal internal friction under moderate deformation. In contrast, at ε_max_ = 1.7, the stress exhibits a pronounced decay immediately after the onset of relaxation (*t*_0_) and gradually approaches a plateau value, as shown in the inset of Figure 4(a). This substantial stress decay is attributed to structural rearrangements within the polymer network, generally believed to arise from chain slippage or bond rupture—mechanisms that are negligible at ε_max_ = 0.7.

**Figure 4:**
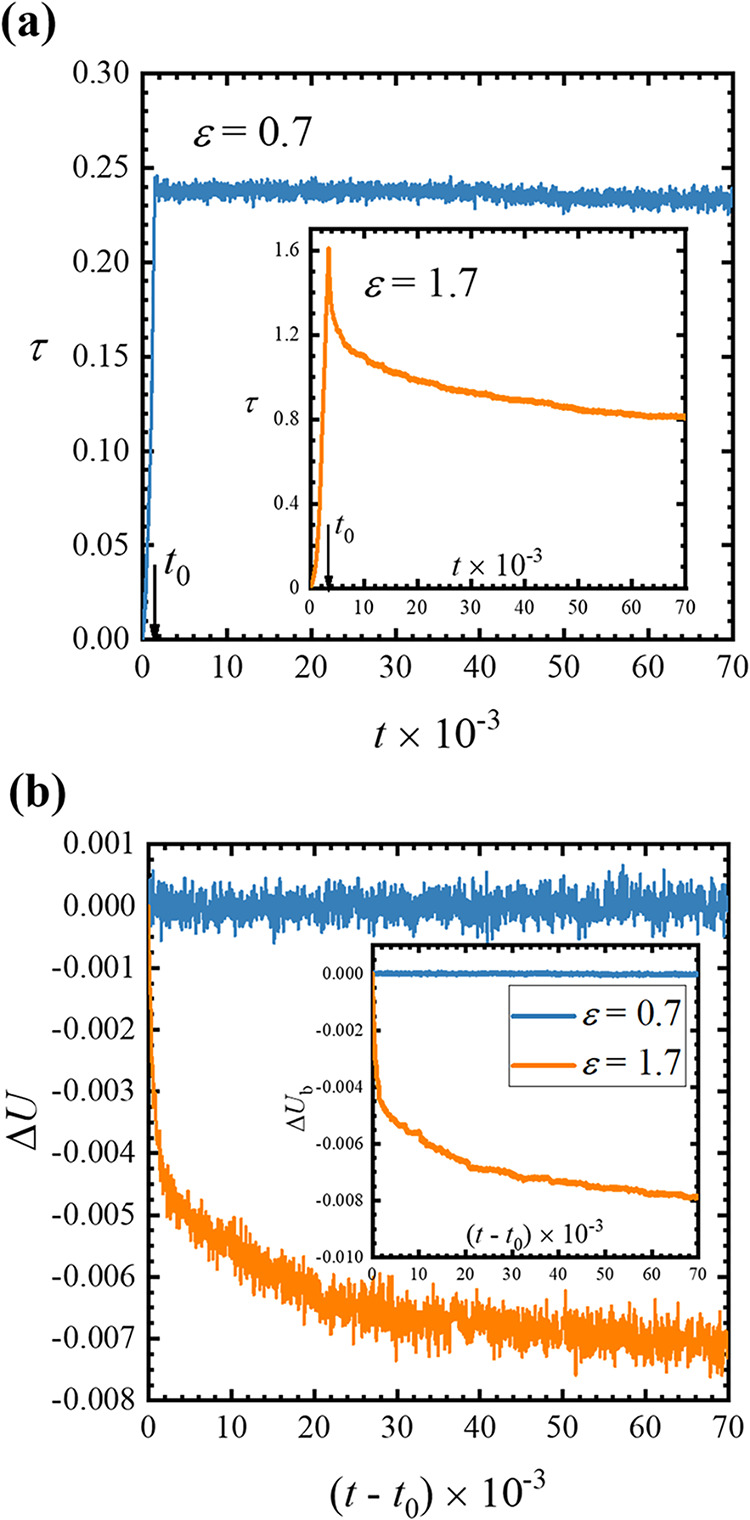
(a) Stress relaxation at fixed strains, shown by the variation of τ over time. The main plot is for ε_max_ = 0.7; the inset shows ε_max_ = 1.7. (b) Variation of the internal energy (Δ*U*) as a function of time (*t*), with the inset showing the corresponding change in bond energy (Δ*U*_b_).

Beyond the macroscopic response, the microstructural origin of stress relaxation can be elucidated by examining the temporal evolution of the internal energy change (Δ*U*), defined relative to its value at the onset of relaxation, *U*(*t*_0_). The total Δ*U* comprises the bond energy (Δ*U*_b_), bending energy (Δ*U*_d_), and nonbonded energy (Δ*U*_n_), each representing a distinct mode of network adjustment. Figure 4(b) presents the time evolution of Δ*U* after the gel is deformed to ε_max_ = 0.7 or 1.7. At ε_max_ = 0.7, Δ*U* fluctuates only slightly around its initial equilibrium value at *t*_0_, indicating that the network structure remains essentially unchanged during relaxation. In contrast, at ε_max_ = 1.7, Δ*U* decreases progressively with time, signifying the release of the stored potential energy as the network reorganizes into a lower-energy configuration. Decomposition of Δ*U* reveals that the dominant contribution to this decrease arises from the bond energy term Δ*U*_b_, as illustrated in the inset of Figure 4(b). Since the strain is maintained constant, the reduction in Δ*U*_b_ reflects gradual bond relaxation rather than macroscopic deformation. This finding suggests that stress relaxation at ε = 1.7 originates from microstructural rearrangement involving bond rupture, whereas such events are absent at ε = 0.7, where configurational changes and internal friction are negligible. Direct evidence for the role of bond failure in stress relaxation can be clearly obtained from a single simulation run rather than from the ensemble average over many runs shown in Figure 4(a). As illustrated in Figure S4 of the Supporting Information, each bond-rupture event causes a sudden drop in stress during stress relaxation, accompanied by structural reorganization.

To gain a deeper understanding of the microscopic mechanism of stress relaxation, the variation in the mean length of intact bonds (*l*_b_) and the mean end-to-end distance of intact strands (*S*_ee_) are monitored throughout the relaxation process at ε_max_ = 1.7. As shown in Figure 5(a), *l*_b_ increases during stretching and reaches a maximum at *t*_0_, where *t*_0_ denotes the onset of the relaxation period. Thereafter, *l*_b_ gradually decreases under the fixed strain ε_max_ = 1.7, exhibiting a relaxation behavior consistent with the macroscopic stress response (see Figure 4(a)). The mean end-to-end distance of intact strands is determined by measuring the spatial distance between beads connected to two distinct cross-linkers along the same polymer chain. The inset of Figure 5(a) further reveals that the mean strand length exhibits a relaxation behavior similar to that after *t*_0_, progressively decreasing over time. These results indicate that relaxation occurs cooperatively at both the bond and strand levels, where microscopic structural units within the network relax in a coordinated manner, leading to a gradual reconfiguration of the internal network structure.

**Figure 5:**
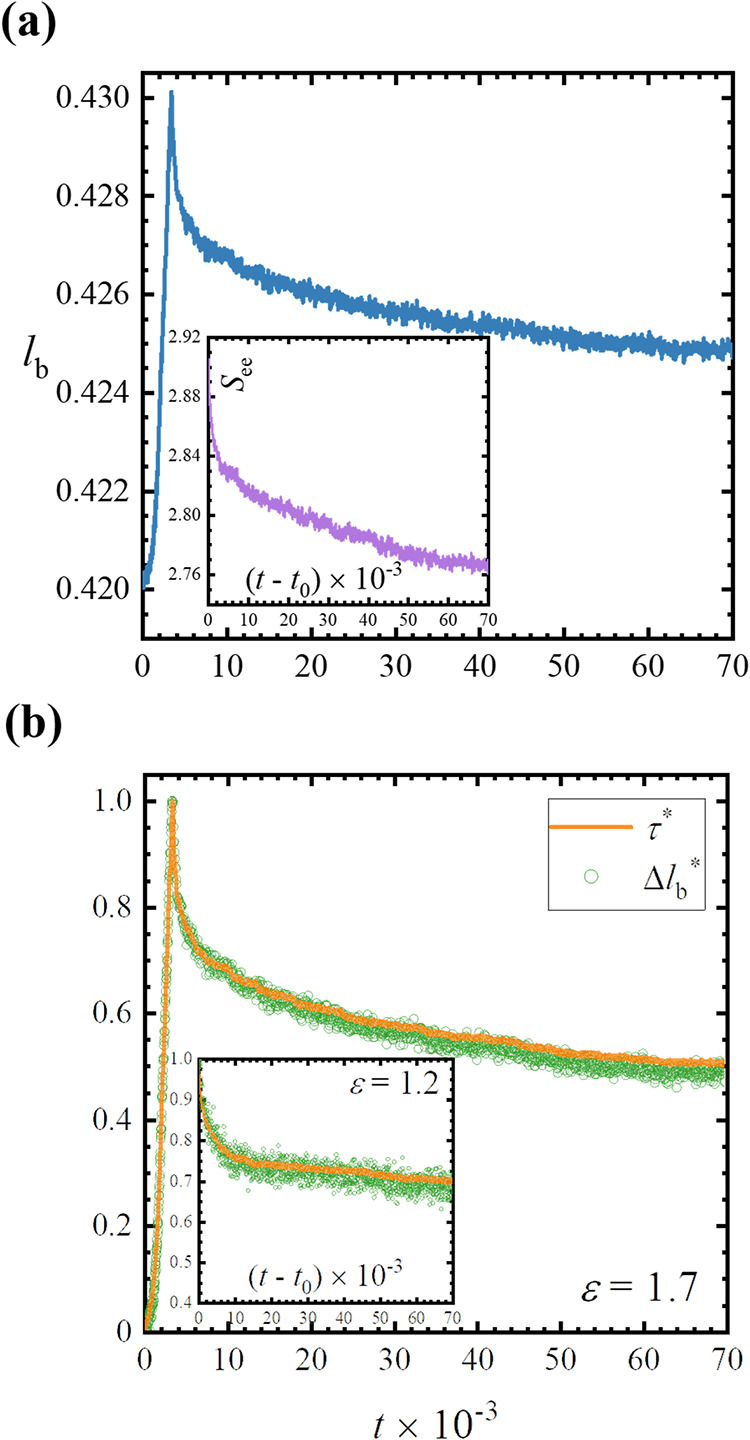
(a) Time evolution of the mean bond length (*l*_b_) during stress relaxation at a fixed strain ε_max_ = 1.7. The inset shows the corresponding time evolution of the mean strand length (*S*_ee_) after *t*_0_ during the relaxation process. (b) Time evolution of the normalized bond-length change (Δ*l*_b_^*^) and normalized stress (τ*) at ε_max_ = 1.7; the inset shows the corresponding curves at ε_max_ = 1.2.

To enable a direct comparison between microscopic and macroscopic relaxation behaviors, Figure 5(b) displays the time evolution of the normalized bond length (Δ*l*_b_^*^) and normalized stress (τ*) at ε_max_ = 1.7 and ε_max_ = 1.2. Here, Δ*l*_b_^*^ is defined as Δ*l*_b_^*^ = [*l*_b_(*t*) – *l*_b_(0)]/[*l*_b_(*t*_0_) – *l*_b_(0)] and the stress relaxation ratio τ* is defined as τ* = τ­(*t*)/τ­(*t*_0_), where *l*_b_(0) represents the mean bond length at the initial equilibrium state. At ε_max_ = 1.7, τ* stabilizes near 0.5 eventually, indicating that roughly half of the initial stress relaxes over time. In contrast, at ε_max_ = 1.2, τ* levels off around 0.7, implying a smaller degree of relaxation. When Δ*l*_b_ and τ are normalized to unity by their respective maximum values, the resulting Δ*l*_b_^*^ and τ* curves nearly coincide, indicating a strong correlation between microscopic bond relaxation and the macroscopic stress response. These findings indicate that the stress relaxation of an ideal elastomer is governed primarily by bond-level dynamics with minimal contribution from internal friction, although both mechanisms can ultimately lead to large-scale conformational reorganization.

To further elucidate the microscopic origin of stress relaxation under large deformation, the distributions of the end-to-end distance of intact strands (*S*_ee_) and the bond length (*l*_b_), denoted as *P*(*S*_ee_) and *P*(*l*_b_), are analyzed at three representative stages: before stretching (*t* = 0), at the onset of relaxation (*t* = *t*_0_ = 3400), and after relaxation (*t* = *t*_f_ = 7 × 10^4^). As shown in Figure 6(a), *P*(*S*_ee_) shifts slightly toward larger values upon stretching, reflecting the extension of polymer strands under deformation. During the subsequent relaxation process, the distribution at *t* = *t*_f_ remains largely similar to that at *t* = *t*_0_, exhibiting a slight decrease in the population of large *S*_ee_ and a corresponding increase in small *S*_ee_, indicative of minor structural relaxation. The inset of Figure 6(a) shows the corresponding *P*(*l*_b_) distributions, which are generally similar to one another, displaying a slight rightward shift upon stretching and a slight leftward shift after relaxation. During the relaxation process from *t*_0_ to *t*_f_, the macroscopic stress relaxation ratio reaches as high as 0.5, and both the mean *S*_ee_ and mean *l*_b_ decrease noticeably. However, the variations in *P*(*S*_ee_) and *P*(*l*_b_) remain minor, suggesting that the network microstructure undergoes only a limited rearrangement despite substantial macroscopic stress relaxation.

**Figure 6:**
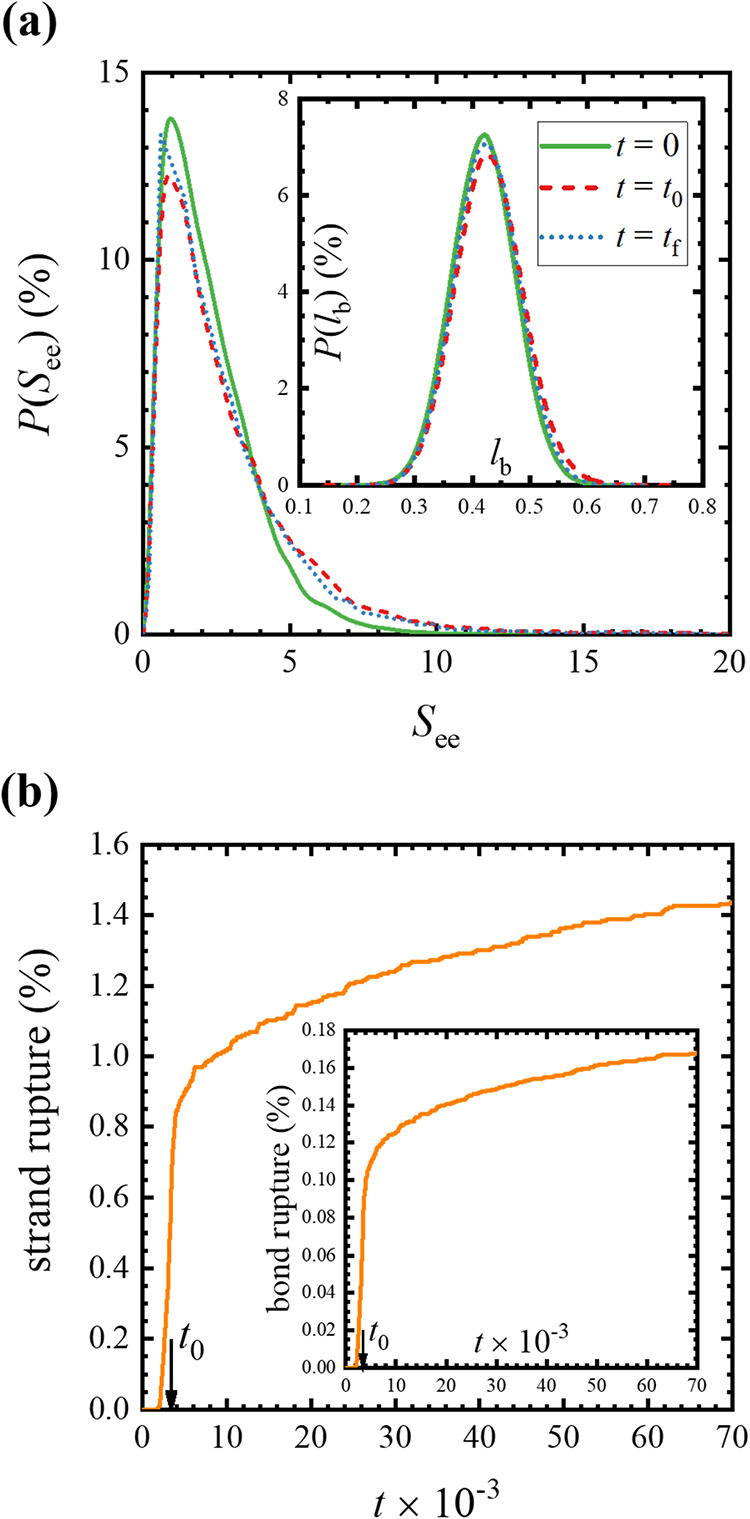
(a) Distributions of the end-to-end distance of intact strands at three representative stages: before stretching (*t* = 0), at the onset of relaxation (*t* = *t*_0_), and after relaxation (*t* = *t*_f_). The inset presents the corresponding bond-length distributions. (b) Time evolution of the strand rupture ratio (%), with the inset showing the corresponding bond-rupture ratio.

Since the overall strand and bond distributions exhibit only minor variations, their rupture statistics can serve as a more sensitive indicator of microscopic structural changes. Figure 6(b) presents the evolution of the rupture ratios for both strands and bonds, defined as the fraction of broken elements relative to their initial totals. In the early stage of stretching (ε ≈ 0.9), no rupture is observed, indicating that the network deforms elastically. Once the strain exceeds this threshold, rupture events emerge and increase progressively with further stretching. During stress relaxation, the rupture ratio continues to increase under the fixed-strain condition, though at a much slower rate, suggesting that a small number of rupture events still occur even after the macroscopic deformation is held constant. Nevertheless, the overall rupture ratios remain low, below 1.5% for strands and 0.17% for bonds, indicating that these breakages are rare events. Despite their scarcity, such limited rupture events play a crucial role in driving overall stress relaxation and internal reconfiguration of the network.

### 3.3 Stress Relaxation: Tensile Strand Failure

To identify where rupture events are most likely to occur, the strand stretch ratio, α_s_ = *S*_ee_/*S*_c_, is analyzed, where *S*_c_ is the contour length of each strand, obtained by summing the bond lengths along that strand, and varies with time. The maximum value of α_s_ = 1 corresponds to a fully extended strand. Figure 7(a) compares the distributions of α_s_, denoted as *P*(α_s_), at equilibrium (*t* = 0) and at the onset of relaxation (*t* = *t*_0_, ε = 1.7). At equilibrium, the average stretch ratio is 0.665, indicating that many strands already adopt semiextended conformations. The temporal evolution of the mean strand stretch ratio is shown in the inset of Figure 7(a). During stretching, α_s_ rises sharply with strain and reaches its maximum value at *t*_0_. At this point, the mean value increases to 0.742, while *P*(α_s_) shifts rightward toward α_s_ = 1; its peak position moves from α_s_ ≈ 0.80 to α_s_ ≈ 0.93, revealing that a larger fraction of strands becomes highly stretched under the applied deformation. The rightward shift of the distribution suggests that an increased fraction of strands is subjected to tensile deformation, potentially corresponding to regions in which rupture events preferentially occur.

**Figure 7:**
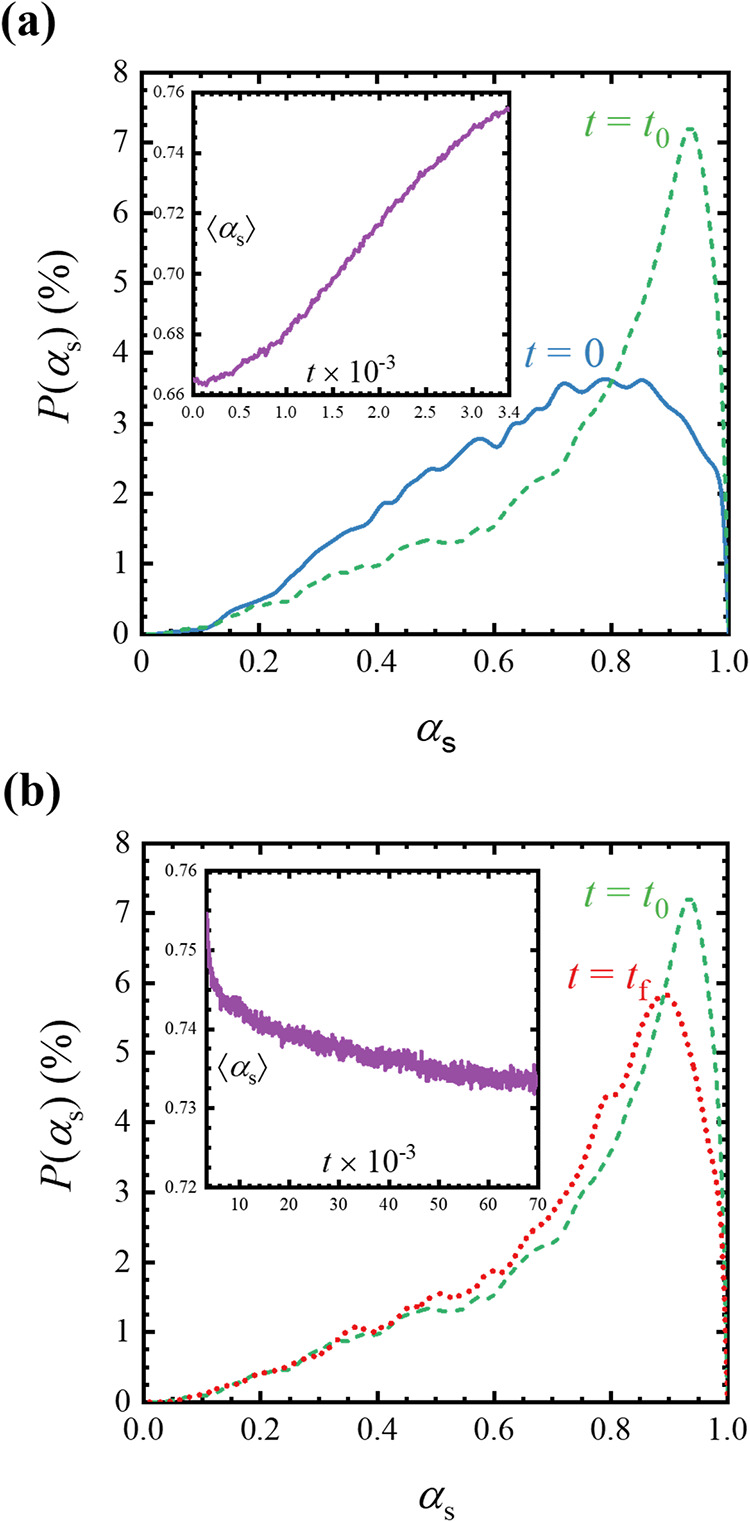
(a) Distributions of the strand stretch ratio (α_s_) at equilibrium (*t* = 0) and at the onset of relaxation (*t* = *t*_0_). The inset shows the temporal evolution of the mean strand stretch ratio during stretching. (b) Distributions of α_s_ at *t*_0_ and *t* = *t*_f_, showing a slight leftward shift after relaxation. The inset shows the temporal evolution of ⟨α_s_⟩ during relaxation.

Note that a distribution *P*(α_s_) biased toward higher values at *t* = 0 does not imply that the strands are already under tension in the absence of an applied load. A high value of α_s_ may simply indicate that a polymer chain, originally in a coiled conformation, has become highly extended without any bond stretching. In other words, a distribution *P*(α_s_) shifted toward higher values indicates that a substantial fraction of strands are in a highly extended state due to cross-linking-induced confinement, rather than significant bond stretching. Indeed, a comparison of the bond-length distribution of the network at *t* = 0 with that of free polymer chains without cross-linking shows that their bond-stretching states are essentially the same, as evidenced by the nearly indistinguishable *P*(*l*_b_) distributions. In reality, bonds in an equilibrium state may indeed fail solely because thermal fluctuations allow them to overcome the energy barrier. However, such events are generally not observed experimentally because they are rare and typically occur on time scales far beyond those accessible in experiments. A similar consideration applies to the simulations. Accordingly, bond-rupture events are not observed in the equilibrium networks in our simulations. In this respect, our simulation model is consistent with reality.

Following the strand stretch ratio distribution analysis in Figure 7(a),(b) compares the distributions of α_s_ at *t*_0_ and *t*_f_, corresponding to the onset and completion of stress relaxation. The inset of Figure 7(b) shows the temporal evolution of ⟨α_s_⟩, which gradually decreases from 0.755 at *t*_0_ to 0.733 at *t*_f_, indicating that strand-level relaxation occurs even under fixed-strain conditions. After relaxation, *P*(α_s_) shifts slightly leftward, though it remains right-shifted relative to *P*(α_s_) at *t* = 0. This leftward shift implies that some highly stretched strands fail during the relaxation process, thereby reducing the mean stretch ratio of the network and contributing to the observed macroscopic stress relaxation. These results further suggest that even a small number of failure events (Figure 6(b)) occurring in highly stretched strands can effectively release local stress and facilitate structural reorganization within the network.

To directly examine how failure events correlate with stretched strands during stress relaxation, Figure 8(a) presents the short-time evolution of the strand stretch ratio α_s_(*t*) over the final ≈1.0 time unit preceding each failure event, in contrast to the overall relaxation process that spans 70,000 time units. Each representative curve corresponds to an individual strand that is about to fail. Although thermal fluctuations are present, these strands consistently maintain large stretch ratios, and their ensemble-averaged curve remains above α_s_ ≈ 0.95 during the final 1.0 time unit before failure. This shows that failure predominantly occurs in strands that are already highly extended and subjected to a strong tensile loading. The inset presents α_s_(t) for some intact strands that do not fail within the same observation window. In this case, the individual curves remain noticeably lower than those of the failing strands, reflecting more relaxed, loosely extended conformations. The clear separation between intact, loosely stretched strands and failing, highly stretched strands confirms that failure is localized to a small subset of strongly stretched strands, rendering failure effectively deterministic at the strand level.

**Figure 8:**
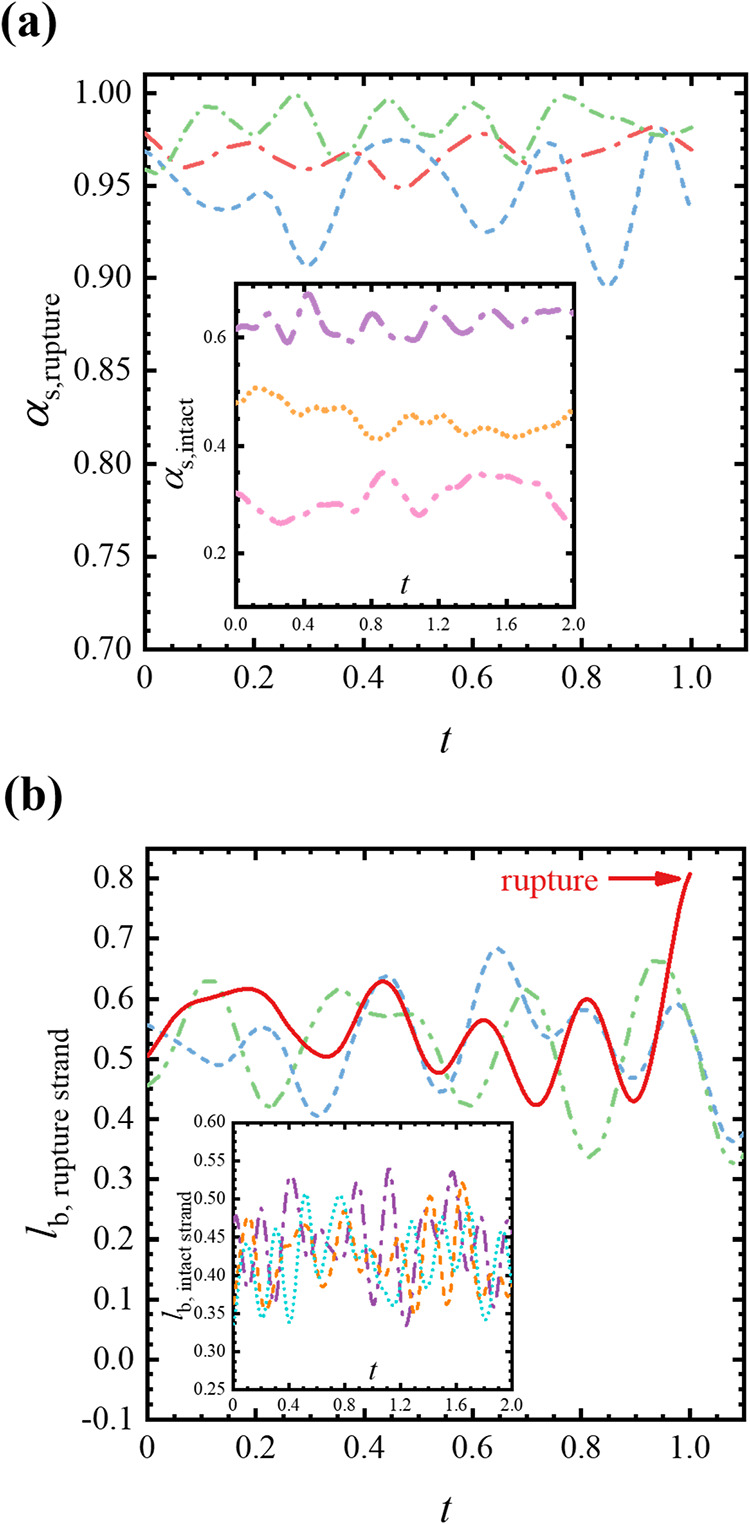
(a) Fluctuations of the strand stretch ratio α_s_ for three strands that fail shortly afterward. The inset shows α_s_ for three intact strands that do not fail over the same time window. (b) Bond-length fluctuations of three bonds within a single highly stretched strand that soon ruptures. The inset shows bond-length fluctuations of three bonds within intact strands over the same time window.

Complementary to the strand-level analysis, Figure 8(b) resolves the bond-length dynamics within a single highly stretched strand that is about to fail. For this soon-to-failure strand, the colored curves show the short-time evolution of several individual bond lengths, while the red curve highlights the specific bond that ultimately breaks. All bonds in this tensile strand exhibit pronounced thermal fluctuations, with their lengths oscillating between 0.4 (below the equilibrium value) and 0.7 (slightly below the critical length) prior to rupture. Notably, the bond that eventually breaks is not necessarily the most stretched one at earlier times. Instead, rupture occurs only when a fluctuation drives that bond to a sufficiently large extension, consistent with a thermally activated process that surmounts a stress-lowered energy barrier rather than deterministic mechanical failure triggered by exceeding a critical strain. The inset shows the bond-length fluctuations within an intact strand over the same time window. In this case, all representative bonds fluctuate around more moderate extensions (≈0.35–0.55), indicating that bonds in intact strands never reach the highly stretched states required to trigger rupture. The mean bond length of the intact strand is approximately 0.427, which is significantly lower than that of the soon-to-failure strand (0.532).

For an ideal hydrogel elastomer in which friction is negligible, stress relaxation under fixed strain is enabled by thermally activated bond-rupture events that occur preferentially in tensile strands that remain highly stretched. As shown in Figure 8, strands that are about to break are already highly stretched prior to rupture, while the bonds within these strands exhibit pronounced thermal fluctuations. Rupture occurs only when a rare fluctuation supplies sufficient thermal energy to drive a bond to a large extension and surmount the stress-lowered energy barrier rather than simply breaking the most highly stretched bond in a deterministic manner. Each rupture event locally releases stress and permits the surrounding network to reorganize into a more relaxed configuration under the same macroscopic strain, thereby contributing to the gradual loss of stress. Taken together, these results show that while rupture is strand-selective and thus effectively deterministic at the level of highly stretched strands, the choice of which specific bond ruptures within a tensile strand is intrinsically stochastic, governed by rare thermal fluctuations.

### 3.4 Creep: Strand Length Evolution and Bond-Rupture Dynamics

Similar to our analysis of the microscopic mechanism of stress relaxation, the microscopic origin of creep can also be investigated by using mesoscale simulations. To probe the creep response of the present network, stress-controlled simulations are performed at five stress levels, corresponding to initial strains ε*_i_* between 0.8 and 1.5. Figure 9(a) plots the strain increment Δε­(*t*) = ε­(*t*) – ε*_i_* as a function of time after the stress is fixed. For the lowest stress τ = 0.36 (ε*_i_* = 0.8), which lies in the elastic regime, Δε­(*t*) fluctuates around zero with only a very slight increase over long times, indicating negligible creep within the observation window. By contrast, for the two higher stresses, τ = 0.61 (ε*_i_* = 1.0) and τ = 0.92 (ε*_i_* = 1.2), Δε­(*t*) increases gradually with time, and two distinct regimes can be identified. As illustrated in the right inset, Δε initially rises rapidly, corresponding to primary creep, but the rate of increase slows for *t* > 300, indicating the onset of secondary creep. Nonetheless, within the present simulation time window, no transition to a tertiary creep regime is observed. The left inset shows the corresponding stress histories, confirming that the applied stresses remain essentially constant throughout the tests.

**Figure 9:**
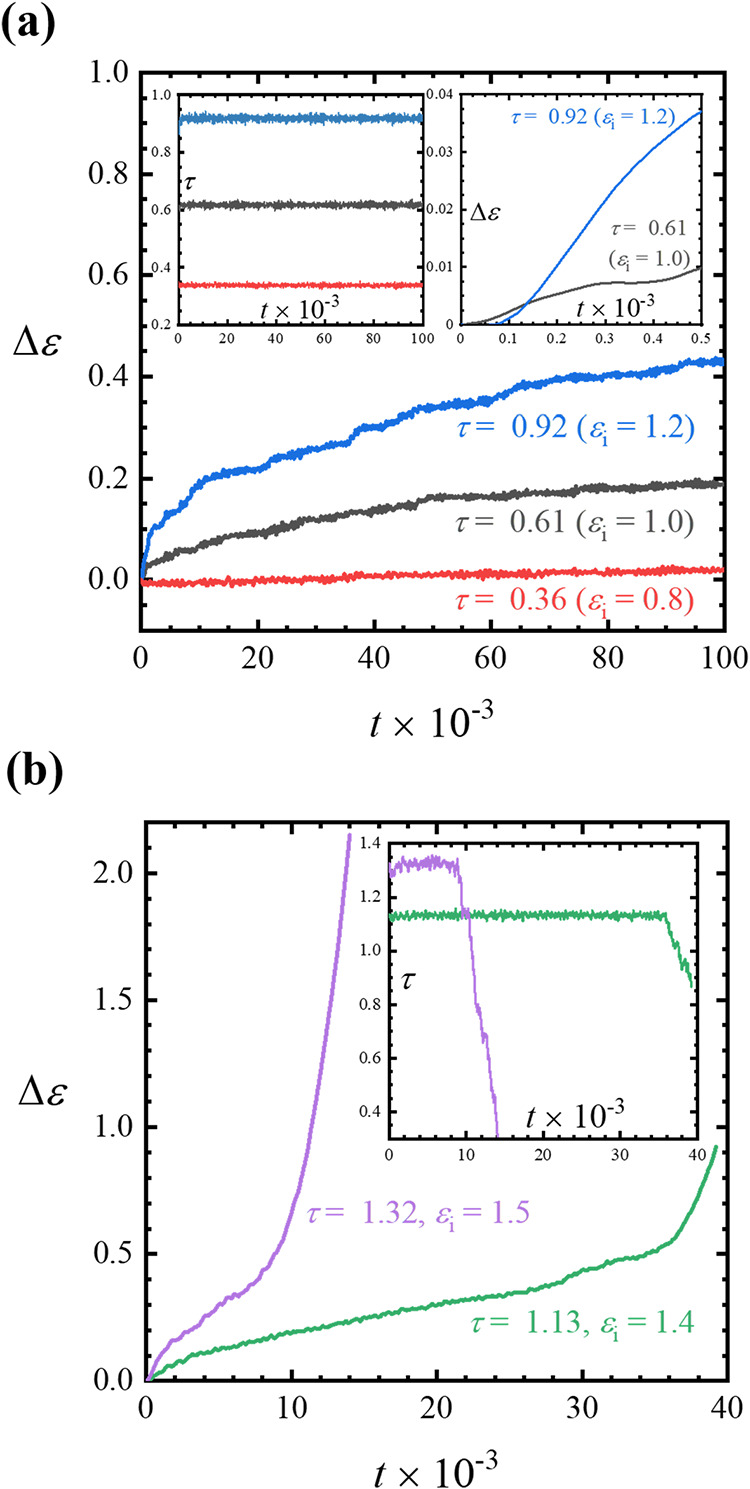
Strain increment as a function of time after the stress is fixed at different levels of τ. The insets present the corresponding stress–time responses. (a) τ = 0.36, 0.61, 0.92. (b) τ = 1.13, 1.32. The right-hand-side inset in panel (a) shows a magnified view of the initial strain increment.

Figure 9(b) shows the creep response at two higher applied stresses, τ = 1.13 (ε*_i_* = 1.4) and 1.32 (ε*_i_* = 1.5). Unlike the behavior at lower stresses (Figure 9(a)), the strain evolution in both cases clearly exhibits the three characteristic creep regimes: an initially decelerating primary creep, a quasi-steady-state secondary creep, and finally an accelerating tertiary creep that ends in rupture. As the applied stress approaches the tensile strength, the secondary creep rate increases and the tertiary stage develops more rapidly, leading to a shorter time to failure; the sample with τ = 1.13 fails at *t* ≈ 40 × 10^3^, whereas that with τ = 1.32 fails at *t* ≈ 15 × 10^3^. The inset displays the corresponding stress–time curves, showing that the applied stress remains essentially constant throughout the primary and secondary regimes but drops sharply at failure due to the rapid elongation during the tertiary stage. In other words, the constant-stress condition can no longer be maintained once the tertiary stage sets in. These behaviors are qualitatively consistent with experimental observations of creep-to-failure under nominally constant stress.

To elucidate the microscopic origin of the primary and secondary creep regimes, we examined the time evolution of the mean strand and bond lengths under identical loading conditions. Figure 10(a) shows the variation of the mean end-to-end length ⟨*S*_ee_⟩ of intact strands as a function of time during a creep test at *ε*_i_ = 1.4. Immediately after the application of a constant stress, ⟨*S*_ee_⟩ initially increases rapidly, then grows more slowly, and finally accelerates into a sharp decrease, closely mirroring the strain evolution during creep: a decelerating primary regime, a quasi-steady secondary regime, and a tertiary stage culminating in failure (Figure 9). This behavior suggests that the creep strain mainly originates from the progressive extension and straightening of intact strands associated with network rearrangement. In contrast, the inset displays the mean bond length, ⟨*l*_b_⟩, which exhibits only small thermal fluctuations around a constant value and shows no systematic drift with time prior to the onset of tertiary creep and failure. The nearly time-independent ⟨*l*_b_⟩ is fully consistent with the imposed constant stress, indicating that the network creeps primarily through gradual strand-level elongation rather than further stretching of individual bonds.

**Figure 10:**
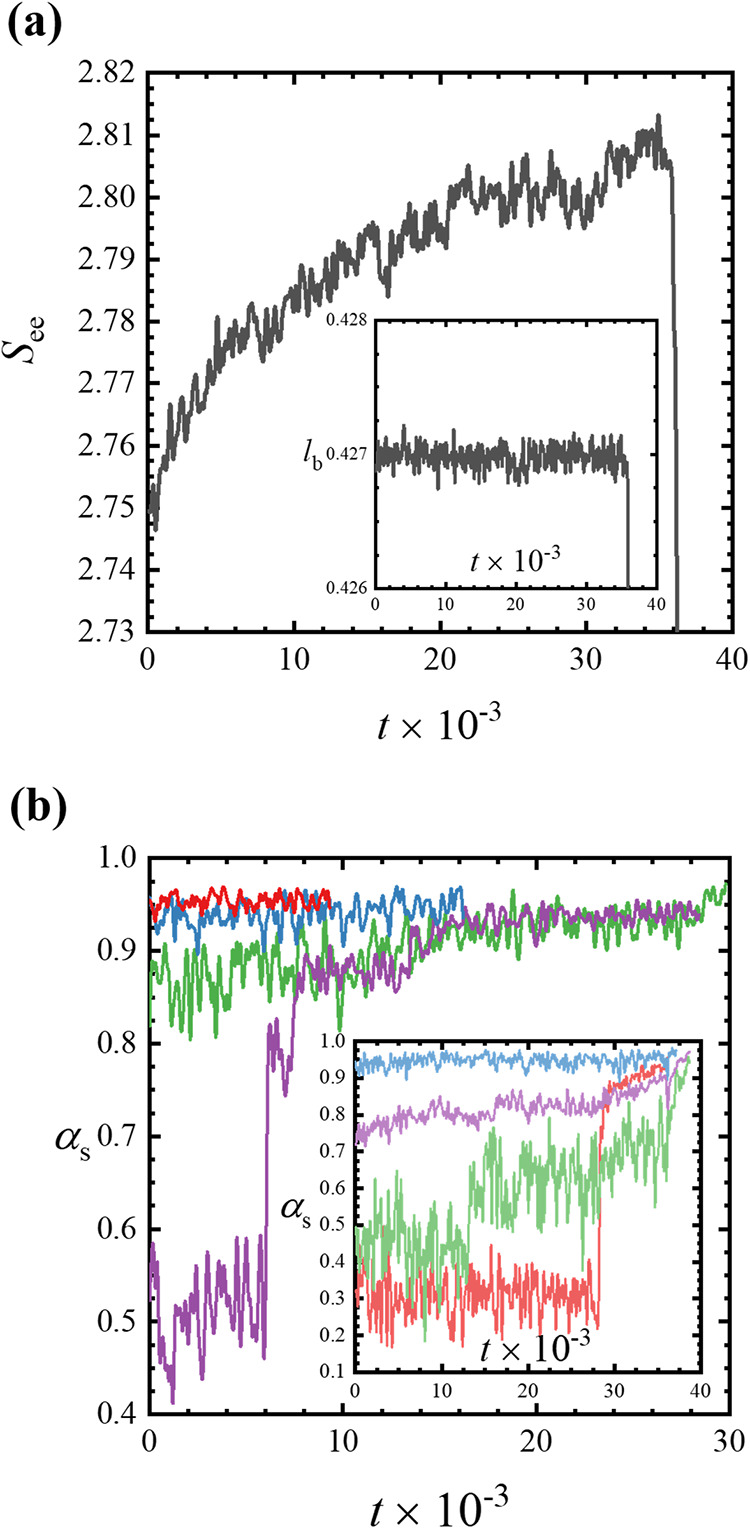
(a) Time evolution of the mean strand length ⟨*S*_ee_⟩ during a creep test at τ = 1.13. The corresponding time evolution of the mean bond length ⟨*l*_b_⟩ is shown in the inset. (b) Time evolution of α_s_ for representative tensile strands that fail in the primary and secondary creep regimes; the inset shows α_s_(*t*) for strands failing in the tertiary regime.

Network rearrangement during creep under constant loading is enabled by crucial bond-rupture events. To identify the strands responsible for rupture, we track the stretch ratios of strands that fail in different creep regimes. Figure 10(b) plots representative α_s_(*t*) for strands that rupture during the creep test at ε*_i_* = 1.4. Note that the stretch ratio of a broken strand is no longer defined. The sudden jumps in α_s_ do not indicate failure of the plotted strand itself but instead reflect abrupt local load redistribution caused by the failure of other nearby load-bearing strands. Strands failing in the primary and secondary creep regimes are shown in the main panel, whereas those failing in the tertiary regime are shown in the inset. The former occurs under higher, approximately constant stress, while the latter is associated with lower but varying stress. In both cases, strands destined to fail may initially have either low stretch ratios (e.g., α_s_ ≈ 0.4) or higher values (α_s_ > 0.8). Nevertheless, regardless of the creep regime, α_s_ increases progressively during creep, and all rupturing strands must reach a highly stretched state with α_s_ exceeding approximately 0.9 prior to failure. This observation indicates that gradual strand straightening and tightening are driven by bond-rupture-induced network reorganization, ultimately bringing intact strands to a highly stretched state where failure occurs and further rearranges the network. Note that although highly stretched strands have a high probability of failure, they do not necessarily fail during the creep test.

To quantify the bond-rupture events responsible for strand failure during creep, Figure 11 plots the cumulative ratio of ruptured bonds as a function of time. The three stress levels that do not enter the tertiary regime within the observation window (τ = 0.36, 0.61, and 0.92) are shown. For the lowest stress, τ = 0.36, bond rupture is almost negligible throughout the entire test. At τ = 0.61 and 0.92, the rupture ratio initially increases relatively rapidly and then grows much more slowly, closely paralleling the transition from primary to secondary creep. Even by the end of the simulations, however, the total fraction of broken bonds remains below 0.15% of all bonds, whereas the total fraction of failed strands exceeds 0.14%. The inset presents the two higher stresses that exhibit a well-developed tertiary regime (τ = 1.13, 1.32). In both cases, the rupture ratio increases rapidly at early times, slows during the secondary stage, and then accelerates sharply once tertiary creep sets in. Although the cumulative fraction of broken bonds remains below 0.35%, the fraction of failed strands exceeds 3.04%. These results demonstrate that rare bond-rupture events in tensile strands nevertheless govern the structural evolution of the network during creep. Comparing Figures 11 with [Fig fig6](b), the bond-rupture rate does not decay during creep because the reduction in the energy barrier remains essentially unchanged (reflected by the constant elongation in Δ*l*_b_), whereas it decreases during stress relaxation because the barrier reduction diminishes over time (reflected by the gradual decrease in Δ*l*_b_).

**Figure 11:**
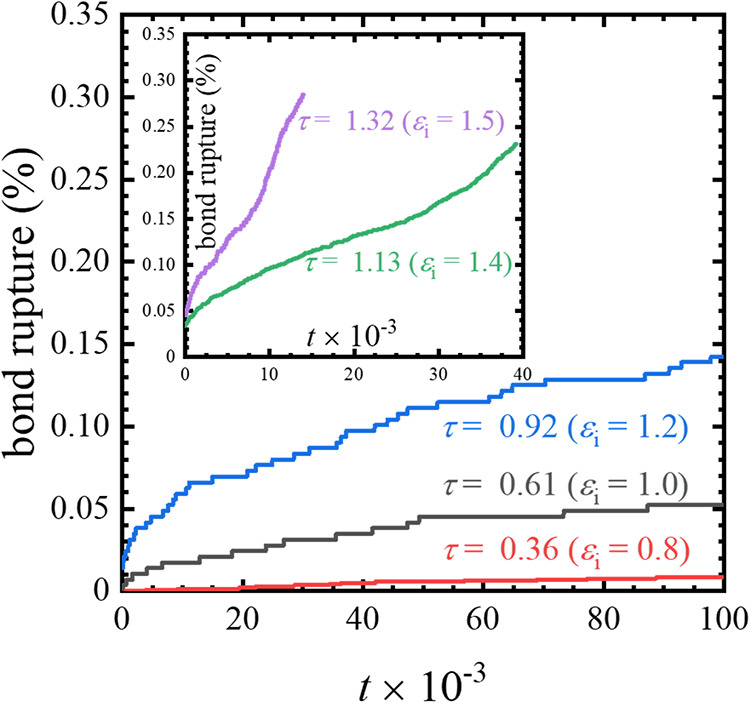
Cumulative ratio of ruptured bonds as a function of time for three lower applied stresses, τ = 0.36, 0.61, and 0.92; the corresponding curves for two higher stresses, τ = 1.13 and 1.32, are shown in the inset.

## 4 Conclusions

In this work, the time-dependent mechanical responses of a hydrogel elastomer film are investigated at the microscopic level using DPD simulations. Under uniaxial stretching, the film exhibits a typical stress–strain behavior, while its elastic and plastic characteristics are further examined through free relaxation. At moderate strains (ε < 0.8), the film shows elastic recoverability, returning essentially to its original configuration after unloading. In contrast, at larger deformations, a small residual strain emerges, signaling the onset of irreversible plasticity. Microscopic analysis further reveals that during free relaxation, the backward evolution of the mean bond length retraces the forward path at moderate strains, whereas the two paths deviate from each other at larger deformations. This microscopic behavior during the loading–unloading process is fully consistent with the macroscopic response reflected in the stress–strain curves. Together, these observations indicate that frictional dissipation, which is absent at the microscopic level, plays a negligible role in the macroscopic cyclic process.

Stress relaxation and creep are essentially absent at moderate strains, consistent with ideal elastomeric behavior, but become pronounced at larger strains. Accompanying the macroscopic stress decay in stress relaxation is the decrease in internal energy dominated by bond-length relaxation. In creep at sufficiently high stress, the mean bond length, similar to the stress, remains essentially constant, whereas the mean strand length, analogous to the strain, evolves through primary and secondary regimes and then accelerates into a tertiary stage associated with failure. Both behaviors can be explained by a unified microscopic mechanism: thermally activated bond rupture occurring within highly stretched tensile strands under a stress-reduced energy barrier, although the specific bond that ruptures within a given tensile strand is not predictable. Although the overall bond-rupture ratio is extremely small, these rare strand-selective events efficiently release stress by reconfiguring the load-bearing network and redistributing tension. The bond-rupture rate decreases during stress relaxation because the reduction in the energy barrier diminishes, whereas it does not decay during creep because the barrier reduction remains essentially unchanged. Although the present study focuses on tensile loading, the proposed mechanism appears to depend on the presence of highly stretched tensile strands rather than on a specific loading path and may therefore also be applicable under compressive or shear loading.

## Supplementary Material



## Notes

Representative snapshots of the hydrogel simulation comparing (a) the equilibrium state and (b) the state under uniaxial deformation (Figure S1); function of the stretch ratio (λ) (Figure S2); additional stress relaxation curves at various imposed strains, with time measured from the onset of relaxation (Figure S3); and bond-rupture events triggering a sudden stress drop, accompanied by structural reorganization (Figure S4) (PDF)
