# Supplementary material for: Thermally Activated Stress Relaxation and Creep in Ideal Hydrogel Elastomers: Rupture of Tensile Strands
Source: ACS Polym Au. 2026 Apr 20;6(3):963–74. doi: 10.1021/acspolymersau.6c00040 (PMC13261728; doi:10.1021/acspolymersau.6c00040)
Supplement: Supplementary file 1 [file lg6c00040_si_001.pdf]

Supporting Information

Thermally Activated Stress Relaxation and Creep in Ideal Hydrogel Elastomers: Rupture of Tensile Strands

Chih-Jung Lin,[1] Yi-Cheng Kao,[2] Heng-Kwong Tsao,[2,] and Yu-Jane Sheng,[1,*]*

[1]Department of Chemical Engineering, National Taiwan University, Taipei 106, Taiwan
[2]Department of Chemical and Materials Engineering, National Central University, Chung-li 320, Taiwan

1. Simulation snapshots

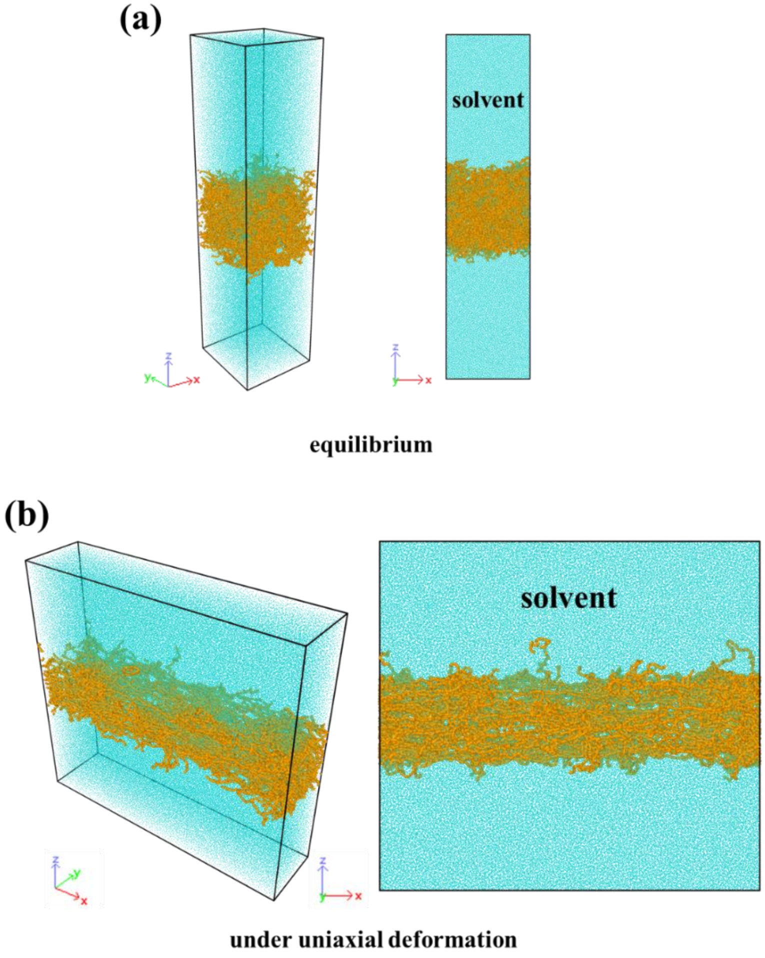

Figure S1. Simulation snapshots (a) at equilibrium and (b) under uniaxial deformation along the *x* direction (ε = 1.7). The hydrogel film is fully immersed between two solvent regions, and periodic boundary conditions are applied in all three directions.

2. **Stress vs. stretch ratio**

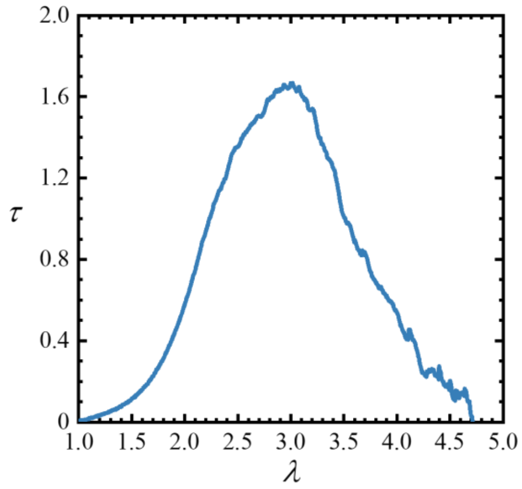

Figure S2. Stress-strain curve replotted as tensile stress τ versus stretch ratio $\lambda = L_x/L_{x0}$. The same overall trend as that in Fig. 1 is observed.

3. **Stress relaxation at multiple imposed strains**

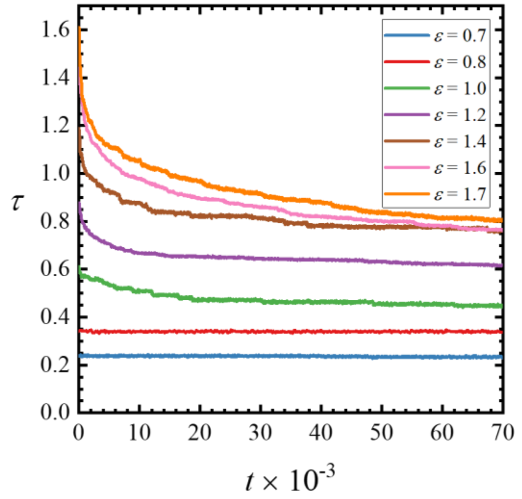

Figure S3. Stress relaxation at multiple imposed strains, showing the time evolution of τ after the prescribed strain is reached and held fixed. Here, $t = 0$ denotes the onset of relaxation.

4. Direct evidence from a representative single run of stress relaxation at ε = 1.7.

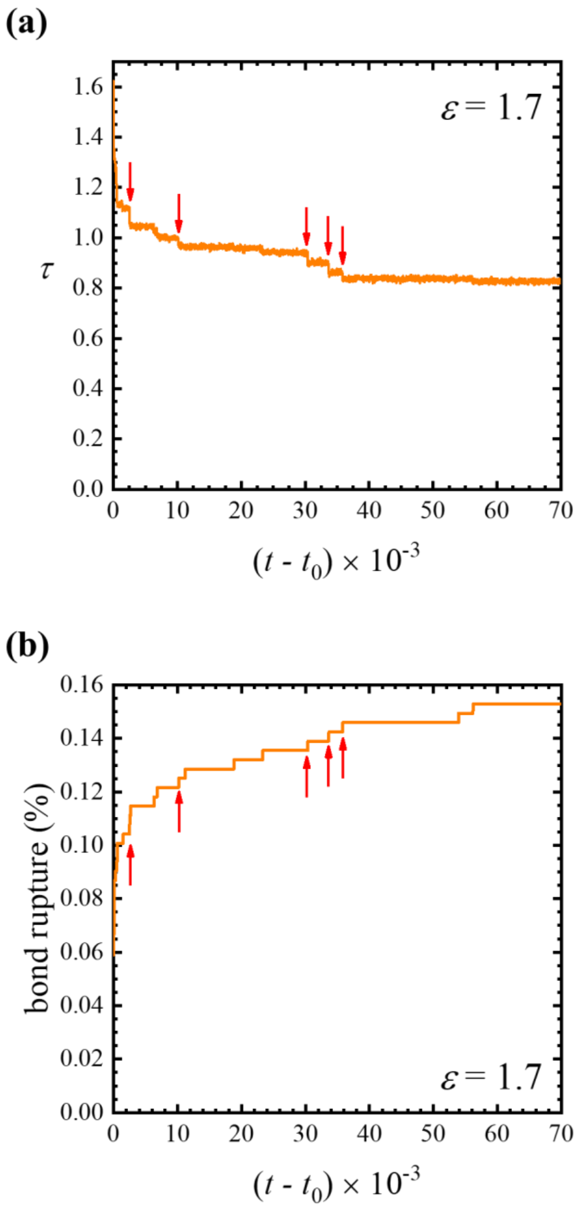

Figure S4. Direct evidence for the role of bond failure in stress relaxation and creep is more clearly seen in a single simulation run at a fixed strain of 1.7 than in the ensemble-averaged results shown in Fig. 4(a). (a) Stress relaxation. (b) Bond rupture events. Each rupture event leads to a sudden stress drop during relaxation, as indicated by the arrows, accompanied by structural reorganization.
